# Use and Out-of-Pocket Costs of Antenatal Fetal Surveillance for Patients With Chronic Conditions

**DOI:** 10.1097/og9.0000000000000136

**Published:** 2026-02-19

**Authors:** Rebecca A. Gourevitch, Amanda Speller, Anna D. Sinaiko, Mark A. Clapp, Jessica L. Cohen

**Affiliations:** Department of Health Policy and Management, University of Maryland, College Park, Maryland; Harvard University Interfaculty Initiative in Health Policy, Boston, Massachusetts; Department of Health Policy and Management and Department of Global Health and Population, Harvard. T.H. Chan School of Public Health, Boston, Massachusetts; and Department of Obstetrics and Gynecology, Massachusetts General Hospital, Boston, Massachusetts.

## Abstract

**OBJECTIVE:**

To measure variation in use and out-of-pocket costs for antenatal fetal surveillance (AFS) among commercially insured patients with chronic hypertension or pregestational diabetes.

**METHODS:**

This was a cross-sectional analysis of pregnancies of at least 20 weeks of gestation between 2017 and 2022. We used administrative enrollment and health insurance claims data from the Health Care Cost Institute, which includes commercially insured pregnancies nationwide. Our sample included patients with chronic hypertension or pregestational diabetes. Our outcomes were the number of days with AFS testing during pregnancy, the out-of-pocket costs for those tests, and those costs as a proportion of all out-of-pocket costs during pregnancy. We used linear regression models to compare variation in these outcomes by health plan type, patient factors (age, clinical characteristics), and geographic factors (rurality, maternity care access, and area-level income and race distributions).

**RESULTS:**

Our sample included more than 150,000 pregnancies with chronic hypertension or pregestational diabetes. Patients with chronic hypertension received a median of five AFS tests, and those with pregestational diabetes received a median of six. There was significant variation in the number of AFS tests received, with 15.9% of patients with chronic hypertension and 12.3% of those with pregestational diabetes receiving no AFS tests during pregnancy. Receiving no AFS was most common for patients in areas that were rural or low income or had a higher concentration of Black individuals. One-quarter of patients with chronic conditions faced out-of-pocket costs for AFS of $264 or more or $301 or more for one-quarter of patients with pregestational diabetes. Patients in point-of-service or preferred provider organization plans, in rural areas, and in areas with a higher concentration of White people had higher costs.

**CONCLUSION:**

Antenatal fetal surveillance is the primary tool for stillbirth prevention, yet we found wide variation in AFS use and costs in this commercially insured population of high-risk pregnancies, including many patients who receive no AFS.

## Abstract 2

This study documents wide variation in use and cost of antenatal fetal surveillance in a commercially insured sample of pregnancies with chronic hypertension or pregestational diabetes.

Stillbirth, a tragic outcome affecting 1 in every 175 births in the United States, has received increasing attention, including a recent working group at the NIH.^1^ One common clinical tool to assess fetal well-being and to estimate stillbirth risk is antenatal fetal surveillance (AFS). Pregnant patients with risk factors for stillbirth such as hypertension and diabetes are recommended to receive regular AFS by the American College of Obstetricians and Gynecologists (ACOG).^2^ The testing frequency varies according to underlying risk, but testing is recommended to occur twice weekly after 32 weeks of gestation for many patients at high risk.^2^

Frequent testing can be costly for patients with commercial insurance because, unlike Medicaid's prohibition on cost sharing for pregnancy-related care, commercially insured patients may incur cost-sharing for some pregnancy-related services.^3,4^ Out-of-pocket costs can be a barrier to utilizing medical care, even when the care is high value.^5,6^ Although some tools exist for helping clinicians and patients discuss costs of care, it is generally challenging to counsel patients on tradeoffs between AFS-associated benefits and costs.^7,8^

Despite the importance of AFS and these potential barriers to care, little work has examined the use of AFS, its out-of-pocket cost among high-risk pregnancies, and how it varies for individuals and across areas. This study examined utilization of AFS, variation in its use by demographics and materntiy care access, and the associated out-of-pocket costs. We focused on patients with chronic hypertension and pregestational diabetes because these conditions are among the most prevalent risk factors for stillbirth, affecting approximately 5% and 1% of pregnancies, respectively, and are diagnosed before the third trimester when most routine AFS is initiated.^9–11^

## METHODS

This cross-sectional study used administrative health enrollment and medical claims data from the Health Care Cost Institute. This database included commercial health insurance claims data from multiple carriers, including more than 55 million fully insured or self-insured members per year (approximately one-third of all people with employer-sponsored insurance).^12^ We used International Classification of Diseases, 10th Revision (ICD-10) diagnosis and procedure codes to identify deliveries (Appendix 1, available online at http://links.lww.com/AOG/E426). We included all deliveries (live births and stillbirths) at 20 weeks of gestation or more between January 1, 2017, and December 31, 2022. We did not restrict the sample on the basis of gestational age at delivery because clinicians have discretion over when to begin AFS using clinical factors that are not observable in our data. We conducted a sensitivity analysis among births delivered at or after 37 weeks of gestation. We calculated the start of each pregnancy using a previously validated algorithm based on ICD-10 codes for gestational age.^13^ The prenatal period for each pregnancy began at the estimated start of each pregnancy and ended the day before the delivery date. We included patients who were continuously enrolled in a health plan in the Health Care Cost Institute data for their entire pregnancy. We excluded pregnancies for which the patient's age was less than 18 years or greater than 54 years at the time of delivery and pregnancies for which the patient's ZIP code was censored in the data.

Our primary groups of interest were patients with chronic hypertension or pregestational diabetes. These conditions facilitated our analysis because they are generally known from the start of pregnancy, their diagnosis is less sensitive to health care access, and there are ACOG guidelines for using AFS for stillbirth prevention for patients with these conditions.^2^ We identified these conditions from a documented diagnosis code (Appendix 2, http://links.lww.com/AOG/E426) at any time during the pregnancy or during delivery, in any position of diagnosis code. Patients with both conditions were included in both groups.

The primary outcomes for this study were the number of days that an AFS tests was performed during pregnancy and the total out-of-pocket costs associated with those tests. Antenatal fetal surveillance was measured for each patient as the distinct number of service dates with any Current Procedural Terminology code for AFS, including biophysical profile, nonstress test, or fetal Doppler (Appendix 3, http://links.lww.com/AOG/E426). We also separately measured the number of each of these three types of AFS that were used. We measured AFS out-of-pocket costs as the sum of any deductible, coinsurance, and copayments on claim lines with Current Procedural Terminology codes for AFS test during pregnancy. We also measured total out-of-pocket costs for all billed services during the pregnancy period to compute the proportion of total pregnancy out-of-pocket spending that was for AFS.

We identified patients who had any of the other 23 conditions included in ACOG's Committee Opinion for Indications for Outpatient AFS using ICD-10 diagnosis codes if they were observed during pregnancy or delivery (Appendix 4, http://links.lww.com/AOG/E426).^2^ We also identified the patient's age category at the time of delivery (18–24, 25–34, 35–44, 45–54 years) because patients at older ages are also recommended to receive AFS.^2^ We measured the length of gestation in weeks and defined patients with preterm births as those who delivered before 37 completed weeks of gestation.

The study data contained administrative enrollment records that allowed us to identify additional health plan and demographic characteristics of interest. We identified each patient's type of health plan (preferred provider organization [PPO], point of service, health maintenance organization [HMO], or other, which includes other types of plans offered by employers, eg, indemnity plans and exclusive provider organization plans^14,15^). Their plan type was of interest because it can affect cost sharing.

Because travel time is a known barrier to accessing prenatal care, especially for patients living farther from health care clinics,^16^ we characterized patients' geographic access to care in two ways. First, we classified their ZIP code of residence as rural or urban using the U.S. Department of Agriculture's Rural-Urban Community Area Codes. We also described access to maternal health care in their county of residence using the March of Dimes' maternity care access measure.^17^ This measure characterized each county as having full, moderate, low, or no access to maternity care. Counties with no access are called maternity care deserts. These categories were based on the number of hospitals and birth centers offering obstetric care (zero in deserts, less than two in low or moderate access, two or more in full access), the number of obstetric clinicians per 10,000 births (zero in deserts, less than 60 in low or moderate access, 60 or more in full access), and the proportion of women 19–54 years of age without health insurance (any in deserts or full access, 10% or more in low access, less than 10% in moderate access).

Finally, we used area-level measures to describe variation by income and race and ethnicity (individual-level income, race, and ethnicity data were not available). We included a measure of race and ethnicity because there are well-documented disparities in access to prenatal care by income and by racial and ethnic groups.^18^ Specifically, we measured economic and racial concentration within patients' ZIP codes using the Index of Concentration at the Extremes.^19^ These metrics used data from the 2015–2019 American Community Survey to describe how each ZIP code nationwide compares with others in its concentration of people from particular groups. For example, the Index of Concentration at the Extremes for race characterized ZIP codes as having higher concentration of Black non-Hispanic individuals (quintile 1) compared with having higher concentration of White non-Hispanic individuals (quintile 5). The Index of Concentration at the Extremes income quintiles ranged from a higher concentration of individuals with low income (quintile 1) to more concentrated individuals with high income (quintile 5).

We first described the percent of patients with each of the above characteristics in the full sample, the chronic hypertension sample, and the gestational diabetes sample. Our next set of analyses described how the number of days with AFS varied by these patient characteristics separately for each chronic disease group. We presented the percent of patients who received no AFS, the median number of AFS tests received, and the interquartile range of the number of AFS tests. To assess whether the median number of AFS tests received was statistically significantly different by patient characteristics, we performed nonparametric Kruskal–Wallace tests. We also used box plots to depict the variation in the number of all AFS tests received, number of biophysical profiles, and number of nonstress tests received between these two chronic condition groups.

Our next set of analyses described variation overall, and by patient characteristics, in out-of-pocket spending on AFS. We used box plots to depict, for each chronic disease group, the variation in out-of-pocket spending by tertile of the number of AFS tests received. We then reported for each chronic condition group how out-of-pocket spending varied by patient characteristic. Specifically, we reported the percent of pregnancy out-of-pocket spending that was for AFS and the median and interquartile range of out-of-pocket spending on AFS. To assess the statistical significance of the differences in median out-of-pocket spending across patient subgroups, we performed nonparametric Kruskal–Wallace tests. We did not run linear regression analyses because of the right-skewed distribution of our outcomes. We also did not run adjusted models because we were not testing specific hypotheses about the joint relationship between the covariates and the outcomes and instead sought to understand how patients' use and costs of AFS varied independently across their observable characteristics.

We conducted these analyses in the full sample and the subgroup with full-term gestations because pregnant women with earlier deliveries have less time to receive AFS. All tests of statistical significance used a two-sided *P* value with a significance threshold of .05. This study was determined exempt by the Harvard T.H. Chan School of Public Health IRB. All analyses were conducted in R 4.1.2.

## RESULTS

Our sample included 1,719,923 deliveries between January 2017 and December 2022, including 99,016 women with chronic hypertension and 51,341 with pregestational diabetes (Table 1; Appendix 5, http://links.lww.com/AOG/E426, gives the full-term sample). Most deliveries were among women ages 25–34 (60.9% overall), and pregnancies with chronic hypertension or pregestational diabetes were more likely to be among older patients (27.3% ages 35–44 years among the full sample vs 37.4% with chronic hypertension and 39.1% with pregestational diabetes). The majority of the sample lived in urban areas (87.6%) and in counties with full maternity care access (87.6%). Most lived in high-income areas (29.2% in the highest Index of Concentration at the Extremes income quintile), although this was less common among the chronic hypertension and pregestational diabetes samples (23.0% and 24.3%, respectively).

**Table 1.** Sample Characteristics

	Full Sample (N=1,719,923)	Chronic Hypertension (n=99,016)	Pregestational Diabetes (n=51,341)
Percent of full sample		5.8	3.0
Maternal age (y)			
18–24	197,055 (11.5)	7,677 (7.8)	3,632 (7.1)
25–34	1,047,786 (60.9)	53,404 (53.9)	27,159 (52.9)
35–44	469,212 (27.3)	36,990 (37.4)	20,095 (39.1)
45–54	5,870 (0.3)	945 (1)	455 (0.9)
Insurance plan type			
Other	47,228 (2.7)	2,485 (2.5)	1,504 (2.9)
HMO	115,247 (6.7)	6,537 (6.6)	3,053 (5.9)
POS	548,696 (31.9)	30,882 (31.2)	16,696 (32.5)
PPO	1,008,752 (58.7)	59,112 (59.7)	30,088 (58.6)
Rurality			
Urban	1,502,606 (87.6)	85,098 (85.9)	44,938 (87.5)
Rural	217,317 (12.6)	13,918 (14.1)	6,403 (12.5)
Maternity care access			
Full	1,507,327 (87.6)	84,491 (85.3)	44,748 (87.2)
Moderate	43,861 (2.6)	2,700 (2.7)	1,224 (2.4)
Low	87,705 (5.1)	6,272 (6.3)	2,907 (5.7)
Maternity care desert	81,030 (4.7)	5,553 (5.6)	2,462 (4.8)
ICE income quintile			
1 (low income)	90,873 (5.3)	7,835 (7.9)	3,860 (7.5)
2	278,794 (16.2)	19,465 (19.7)	9,765 (19)
3	371,530 (21.6)	22,657 (22.9)	11,862 (23.1)
4	475,967 (27.7)	26,323 (26.6)	13,396 (26.1)
5 (high income)	502,759 (29.2)	22,736 (23.0)	12,458 (24.3)
ICE race quintile			
1 (more Black)	166,967 (9.7)	13,846 (14)	7,081 (13.8)
2	384,263 (22.3)	23,485 (23.7)	13,607 (26.5)
3	478,779 (27.8)	25,570 (25.8)	13,392 (26.1)
4	432,008 (25.1)	21,522 (21.7)	10,551 (20.6)
5 (more White)	257,906 (15.0)	14,593 (14.7)	6,710 (13.1)
Length of gestation			
Weeks of gestation, median (IQR)	39 (38, 39)	37 (36, 38)	38 (36, 39)
Full-term birth	1,520,280 (88.4)	69,971 (70.7)	38,314 (74.6)
Preterm birth	199,643 (11.6)	29,045 (29.3)	13,027 (25.4)
Additional AFS indication(s)	—	92,672 (93.6)	48,648 (94.8)

HMO, health maintenance organization; POS, point of service; PPO, preferred provider organization; ICE, Index of Concentration at the Extremes; IQR, interquartile range; AFS, antenatal fetal surveillance.

Values are n (%) unless indicated otherwise.

“Other” insurance plan type may include indemnity plans and exclusive provider organization plans. Rurality is defined based on the U.S. Department of Agriculture's Rural-Urban Commuting Area Codes 2010 classification system using categorization B and the area ZIP code methodology. Maternity care access is a county-level measure constructed by the March of Dimes based on the number of hospitals and birth centers offering obstetric care (zero in deserts, less than two in low or moderate access, two or more in full access), the number of obstetric clinicians per 10,000 births (zero in deserts, less than 60 in low or moderate access, 60 or more in full access), and the proportion of women 19–54 years of age without health insurance (any in deserts or full access, 10% or more in low access, less than 10% in moderate access). ICE measures economic and racial segregation within geographic areas by ZIP code. Race and income measurements are taken from the 2015–2019 American Community Survey 5-year average, and ICE quintiles are calculated with the method from Krieger et al.^19^ Full-term births are those that reach at least 37 completed weeks of gestation; preterm births are those that deliver before 37 completed weeks of gestation. Additional AFS indication(s) measure indicates the presence of any diagnosis code for a condition for which the American College of Obstetricians and Gynecologists recommends antenatal fetal surveillance other than the chronic condition of interest (chronic hypertension or pregestational diabetes).

Patients with chronic hypertension received a median of five AFS tests during their pregnancy, although there was substantial variation (Table 2). Their interquartile range was one to eight, indicating that 25% received no or one AFS test, and 25% received eight or more. Patients with pregestational diabetes received a median of six AFS tests (interquartile range 3–10). The most common AFS type among both chronic condition groups was a nonstress test (median 2, interquartile range 0–5 for chronic hypertension; median 3, interquartile range 0–7 for pregestational diabetes), followed by biophysical profile (median 1, interquartile range 0–5 for chronic hypertension; median 2, interquartile range 0–6 for pregestational diabetes) (Appendices 6 and 7, http://links.lww.com/AOG/E426). Despite the clinical indications, 15.9% of those with chronic hypertension and 12.3% of those with pregestational diabetes received no AFS tests at all during their pregnancy (Table 2; Appendix 8, http://links.lww.com/AOG/E426, gives the full-term sample).

**Table 2.** Use of Antenatal Fetal Surveillance by Chronic Condition and Area Characteristics

	Chronic Hypertension (n=99,016)			Pregestational Diabetes (n=51,341)		
	Percent With No AFS	Median	IQR	Percent With No AFS	Median	IQR
Full sample	15.9	5	1, 8	12.3	6	3, 10
Insurance plan type						
Other	13.6	5	2, 7	—	6	2, 10
HMO	18.1	4	1, 7	—	6	2, 9
POS	15.7	5	1, 8	12.3	6	3, 10
PPO	15.8	5	1, 8	11.9	6	3, 10
Rurality						
Urban	15.7	5	1, 8	12.1	6	3, 10
Rural	17.2	4	1, 8	13.7	6	2, 9
Maternity care access						
Full	15.6	5	1, 8	12.0	6	3, 10
Moderate	14.8	5	1, 8	—	7	3, 11
Low	18.2	4	1, 7	—	6	2, 9
Maternity care desert	17.7	4	1, 7	—	5	2, 9
ICE income quintile						
1 (low income)	17.6	4	1, 7	14.8	6	2, 9
2	17.1	4	1, 8	13.8	6	2, 9
3	16.3	5	1, 8	12.9	6	2, 10
4	15.1	5	2, 8	11.7	6	3, 10
5 (high income)	14.6	5	2, 8	10.4	6	3, 10
ICE race quintile						
1 (more Black)	17.2	4	1, 7	14.8	5	2, 9
2	16.6	4	1, 7	13.2	6	2, 9
3	15.6	5	1, 8	12.0	6	3, 10
4	15.4	5	1, 8	11.1	6	3, 10
5 (more White)	14.6	5	2, 8	10.3	7	3, 11
Additional AFS indication						
None	47.6	1	0, 3	43.6	1	0, 5
Any	13.7	5	2, 8	10.6	6	3, 10

AFS, antenatal fetal surveillance; IQR, interquartile range; HMO, health maintenance organization; POS, point of service; PPO, preferred provider organization; ICE, Index of Concentration at the Extremes.

“Other” insurance plan type may include indemnity plans and exclusive provider organization plans. Rurality is defined based on the U.S. Department of Agriculture's Rural-Urban Commuting Area Codes 2010 classification system using categorization B and the area ZIP code methodology. Maternity care access is a county-level measure constructed by the March of Dimes based on the number of hospitals and birth centers offering obstetric care (zero in deserts, less than two in low or moderate access, two or more in full access), the number of obstetric clinicians per 10,000 births (zero in deserts, less than 60 in low or moderate access, 60 or more in full access), and the proportion of women 19–54 years of age without health insurance (any in deserts or full access, 10% or more in low access, less than 10% in moderate access). ICE measures economic and racial segregation within geographic areas by ZIP code. The race and income measurements are taken from the 2015–2019 American Community Survey 5-year average, and the ICE quintiles are calculated with the method from Krieger et al.^19^ Empty cells were suppressed because of small sample sizes. All differences in the medians within each group were statistically significant, *P*≤.001, with nonparametric Kruskal–Wallace tests.

**Fig. 1.**
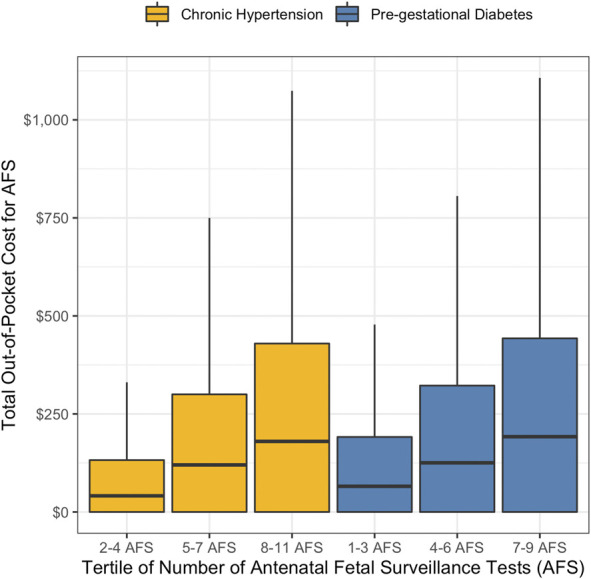
Out-of-pocket costs for antenatal fetal surveillance (AFS) by chronic condition and utilization. Figures show the distribution of out-of-pocket costs by bins of tertiles of AFS utilization for each chronic condition category; each box plot shows the 25th percentile (*bottom line*), median (*middle, bold line*), and 75th percentile (*top line*), as well as the minimum and maximum range (*vertical lines*) of the number of each type of procedure received during pregnancy across each sample. Outliers have been removed for ease of interpretation (individuals with more than nine AFS tests in the chronic hypertension group or with less than two or more than 11 AFS tests in the pregestational diabetes subgroup).

The median out-of-pocket spending on all AFS received during a pregnancy was $61 for patients with chronic hypertension and $80 for patients with pregestational diabetes (Table 3; Appendix 9, http://links.lww.com/AOG/E426, gives the full-term sample). These costs made up 11% of all out-of-pocket spending during the pregnancy period among both groups on average. The interquartile ranges indicate substantial variation, with 25% of patients in both chronic condition groups facing no out-of-pocket costs for AFS, and 25% facing significant costs: $264 or more for patients with chronic hypertension and $301 or more for those with pregestational diabetes. This variation in spending was not only a result of different quantities of AFS used: Figure 1 shows that there was wide variation in out-of-pocket costs among patients who received the same number of AFS tests (Appendix 10, http://links.lww.com/AOG/E426, gives the full-term sample). For example, patients with chronic hypertension who had seven to nine AFS tests during pregnancy had an out-of-pocket spending interquartile range of $0–435, and patients with pregestational diabetes who had 9–11 AFS tests had an out-of-pocket spending interquartile range of $0–462.

**Table 3.** Out-of-Pocket Spending on Antenatal Fetal Surveillance by Chronic Condition and Area Characteristics

	Chronic Hypertension (n=99,016)			Pregestational Diabetes (n=51,341)		
	Percent of OOP Spent on AFS	Median ($)	IQR ($)	Percent of OOP Spent on AFS	Median ($)	IQR ($)
Full sample	10.9	61	0, 264	11.3	80	0, 301
Insurance plan type						
Other	10.8	27	0, 195	11.0	32	0, 228
HMO	8.5	0	0, 87	8.6	0	0, 100
POS	11.2	88	0, 290	12.2	116	0, 347
PPO	11.1	64	0, 269	11.0	78	0, 296
Rurality						
Urban	10.8	60	0, 259	11.1	79	0, 300
Rural	12.0	75	0, 295	12.3	93	0, 340
Maternity care access						
Full	10.9	60	0, 260	11.2	80*	0, 300
Moderate	11.2	61	0, 270	12.0	83*	0, 320
Low	11.1	72	0, 273	11.5	84*	0, 324
Maternity care desert	11.9	81	0, 300	11.4	89*	0, 319
ICE income quintile						
1 (low income)	10.9	60	0, 270	11.3	75	0, 304
2	11.3	68	0, 278	11.6	87	0, 320
3	11.2	69	0, 275	11.4	82	0, 300
4	11.0	61	0, 263	11.3	81	0, 301
5 (high income)	10.3	53	0, 240	10.8	72	0, 287
ICE race quintile						
1 (more Black)	10.0	49	0, 240	10.5	63	0, 276
2	10.5	60	0, 254	10.8	80	0, 297
3	11.0	68	0, 266	11.4	81	0, 304
4	11.4	66	0, 272	11.8	81	0, 314
5 (more White)	11.9	62	0, 291	12.0	86	0, 320
Additional AFS indication						
None	7.3	0	0, 83	5.9	0	0, 92
Any	11.2	70	0, 277	11.6	88	0, 313

OOP, out-of-pocket; AFS, antenatal fetal surveillance; IQR, interquartile range; HMO, health maintenance organization; POS, point of service; PPO, preferred provider organization; ICE, Index of Concentration at the Extremes.

“Other” insurance plan type may include indemnity plans and exclusive provider organization plans. Rurality is defined based on the U.S. Department of Agriculture’s Rural-Urban Commuting Area Codes 2010 classification system using categorization B and the area ZIP code methodology. Maternity care access is a county-level measure constructed by the March of Dimes based on the number of hospitals and birth centers offering obstetric care (zero in deserts, less than two in low or moderate access, two or more in full access), the number of obstetric clinicians per 10,000 births (zero in deserts, less than 60 in low or moderate access, 60 or more in full access), and the proportion of women 19–54 years of age without health insurance (any in deserts or full access, 10% or more in low access, less than 10% in moderate access). ICE measures economic and racial segregation within geographic areas by ZIP code. The race and income measurements are taken from the 2015–2019 American Community Survey 5-year average, and the ICE quintiles are calculated with the method from Krieger et al.^19^ All differences in the medians within each group were statistically significant, *P* ≤ .001, with nonparametric Kruskal–Wallace tests except where noted. Results that were not statistically significant *(P* ≥ 0.05) are indicated with an asterisk.

*Gourevitch. Use and Cost of Antenatal Fetal Surveillance. Obstet Gynecol 2026*.

Use of AFS and out-of-pocket costs varied substantially across patient health plan and demographic characteristics. Table 2 shows that receiving no AFS was more likely among patients in rural areas (17.2% vs 15.7% among those with chronic hypertension, 13.7% vs 12.1% among those with pregestational diabetes), low-income areas (17.6% in lowest Index of Concentration at the Extremes income quintile vs 14.6% in the highest among those with chronic hypertension, 14.8% in the lowest Index of Concentration at the Extremes income quintile vs 10.4% in the highest among those with pregestational diabetes), and areas with a higher concentration of Black individuals (17.2% in the most-Black quintile vs 14.6% in the most-White quintile among those with chronic hypertension, 14.8% in the most-Black quintile and 10.3% in the most-White quintile among those with pregestational diabetes). The distribution of the number of AFS tests received also varied across these demographic characteristics. Patients with full-term deliveries used slightly more AFS tests than the primary study sample (median of five in both samples for those with chronic hypertension; six vs seven among those with pregestational diabetes; Appendix 7, http://links.lww.com/AOG/E426), but the patterns of variation across the full-term sample were similar (see Appendices 6 and 7, http://links.lww.com/AOG/E426).

Out-of-pocket spending on AFS per pregnancy also varied across patient demographic characteristics (Table 3; Appendix 9, http://links.lww.com/AOG/E426, gives full-term sample; Appendix 11, http://links.lww.com/AOG/E426, gives mean spending). Patients enrolled in point-of-service or PPO plans faced higher out-of-pocket spending than those in HMO plans (interquartile range $0–290 for point-of-service or $0–269 for PPO vs $0–87 for HMO among those with chronic hypertension; interquartile range $0–347 for point-of-service or $0–296 for those with PPO vs $0–100 for those with HMO among those with pregestational diabetes). Patients in rural areas faced higher costs than those in urban areas (median $75 vs $60 among those with chronic hypertension, *P*<.001; $93 vs $79 among those with pregestational diabetes, *P*<.001). Spending was also higher among people living in areas with a higher concentration of White people (median $62 in the most-White Index of Concentration at the Extremes quintile vs $49 in the most-Black among those with chronic hypertension, *P*<.001; $86 vs $63 among those with pregestational diabetes, *P*<.001). Variation in out-of-pocket spending was less pronounced by patient income quintiles or maternity care access.

## DISCUSSION

Antenatal fetal surveillance is the primary clinical tool for the assessment of fetal well-being and stillbirth risk mitigation; however, in this analysis of more than 150,000 pregnancies with two common chronic conditions, we found wide variation in AFS use, with substantial proportions of the population receiving fewer AFS tests than recommended by ACOG. For example, despite being recommended to receive one or two AFS tests weekly starting at 32 weeks of gestation,^2^ over 10% of patients in each chronic condition group did not receive any AFS test over the course of pregnancy. This was true even among full-term births.^2^ In addition to chronic hypertension or pregestational diabetes, almost all the patients in the sample (94–95%) had another indication for AFS, underscoring the need for this care. It is important to note that we found that many patients face substantial out-of-pocket costs for AFS, with 25% of patients with chronic hypertension spending over $260 and 25% of patients with pregestational diabetes facing over $300 in AFS costs. Spending was higher among those with PPO or point-of-service insurance plans, patients in rural areas, and patients in areas with a higher proportion of White non-Hispanic individuals.

Prior research has documented the high costs of maternity care and their contributions to unpaid medical bills and financial hardship^3,20–24^ and the higher overall spending associated with chronic conditions during pregnancy.^25,26^ This study adds to the literature by examining the out-of-pocket costs associated with a specific, guideline-based prenatal care service and their variation by patient demographics. Our results highlight that commercial insurance plans, particularly PPO and point-of-service plans, can leave patients vulnerable to high out-of-pocket expenses. In contrast, Medicaid prohibits cost sharing for any pregnancy-related services, leading those patients to face lower costs.^3^ Although the median out-of-pocket costs we observe were under $100, research from other health care settings has shown that even very low out-of-pocket costs can be a barrier to the use of high-value health care.^5,6^ Although we are not able to make causal claims about whether these costs are limiting AFS use in our sample, the prior literature raises this concern.

These costs are just one of many potential barriers to receiving AFS. Though our analysis cannot identify the primary barriers to testing, these gaps in care can occur if their indications are not recognized by clinicians, the clinicians choose not to recommend AFS, or patients decline testing. In addition, patients may not have access to AFS at their regular prenatal care clinic and therefore may need to travel farther to receive AFS. Indeed, we found that patients in rural areas, maternity care deserts, or areas with low access were less likely to receive AFS. We also found that patients living in areas with lower income or with a higher concentration of Black individuals were less likely to receive AFS; this is consistent with prior work finding lower guideline-based prenatal care receipt in low-income areas and those with a high proportion of minoritized individuals.^27^ These results raise concerns about how access to and use of AFS could contribute to racial disparities in stillbirth: Black non-Hispanic people and Native Hawaiian/Other Pacific Islander people experience over twice the rate of stillbirth as White non-Hispanic people.^28^

Our study has limitations. First, our data include only people with commercial insurance and therefore may not generalize to births covered by Medicaid. Although it is important to understand variation in the use of AFS in the Medicaid population, Medicaid exempts all pregnancy-related services from cost sharing, so we would not expect this population to face costs for this care. Second, we cannot observe individual-level race, ethnicity, or income and must therefore rely on ZIP code–level measures, which may be subject to the ecologic fallacy. Third, we lack data on patient-paid health plan premiums; therefore, our results do not reflect total patient spending. Plans with higher cost sharing have lower premiums than other plans; this difference in premiums will therefore offset some of the differences in out-of-pocket spending, but we are unable to measure the magnitude of the offset. Finally, we rely on diagnosis codes to identify comorbidities and gestational age at delivery. Although diagnosis codes for gestational age have been validated as highly accurate,^13^ it is possible that we miss some relevant comorbidities that are not documented in the clinical records. Relatedly, we do not observe pharmaceutical claims for the full sample and are therefore unable to measure whether the blood pressure of each patient with chronic hypertension was controlled with medication.

This study provides novel population-level evidence on variation in the use of AFS and associated costs. It adds to the growing literature that moves beyond traditional prenatal care quality metrics of timing of initiation and adequacy and examines whether patients receive guideline-recommended care.^27,29,30^ Our results highlight the importance of examining use of specific prenatal services, which prior work found can vary regardless of the number of prenatal visits received.^27^ The wide variation in the number of AFS tests and their costs is an important finding underscoring heterogeneity in patients' use and experiences of these tests, identifying which patient populations that may be a higher need for strategies to improve access to AFS. To increase access to this important testing, policy makers are considering reducing patient cost sharing for pregnancy-related care.^31^ Advances in remote fetal monitoring, including for nonstress tests, may also increase patient access but may carry additional costs.^32,33^ The ACOG released its guidelines for outpatient AFS during the study period. Future work should investigate whether these guidelines have led to more consistent use of AFS among patients with the specified indications and the causes and consequences of variation in use of these procedures.

We find wide variation in use and cost of AFS in a commercially insured sample of pregnancies with chronic hypertension or pregestational diabetes. A substantial number of these patients use less AFS than recommended and face hundreds of dollars of costs for this care. It is important for future work to investigate the drivers of this variation in utilization and costs, particularly distance from health care clinics, and their implications for health outcomes.
